# Gelatin Hydrogel Enhances the Engraftment of Transplanted Cardiomyocytes and Angiogenesis to Ameliorate Cardiac Function after Myocardial Infarction

**DOI:** 10.1371/journal.pone.0133308

**Published:** 2015-07-17

**Authors:** Kazuaki Nakajima, Jun Fujita, Makoto Matsui, Shugo Tohyama, Noriko Tamura, Hideaki Kanazawa, Tomohisa Seki, Yoshikazu Kishino, Akinori Hirano, Marina Okada, Ryota Tabei, Motoaki Sano, Shinya Goto, Yasuhiko Tabata, Keiichi Fukuda

**Affiliations:** 1 Department of Cardiology, Keio University School of Medicine, 35 Shinanomachi Shinjuku-ku, Tokyo, Japan; 2 Department of Cardiovascular Surgery, Keio University School of Medicine, 35 Shinanomachi Shinjuku-ku, Tokyo, Japan; 3 Department of Biomaterials, Field of Tissue Engineering, Institute for Frontier Medical Sciences, Kyoto University, 53 Kawahara-cho Shogoin, Sakyo-ku, Kyoto, Japan; 4 Department of Medicine (Cardiology), Tokai University School of Medicine, 143 Shimokasuya, Isehara, Kanagawa, Japan; 5 Japan Society for the Promotion of Science, Tokyo, Japan; Niigata University Graduate School of Medical and Dental Sciences, JAPAN

## Abstract

Cell transplantation therapy will mean a breakthrough in resolving the donor shortage in cardiac transplantation. Cardiomyocyte (CM) transplantation, however, has been relatively inefficient in restoring cardiac function after myocardial infarction (MI) due to low engraftment of transplanted CM. In order to ameliorate engraftment of CM, the novel transplantation strategy must be invented. Gelatin hydrogel (GH) is a biodegradable water-soluble polymer gel. Gelatin is made of collagen. Although we observed that collagen strongly induced the aggregation of platelets to potentially cause coronary microembolization, GH did not enhance thrombogenicity. Therefore, GH is a suitable biomaterial in the cell therapy after heart failure. To assess the effect of GH on the improvement of cardiac function, fetal rat CM (5×10^6^ or 1x10^6^ cells) were transplanted with GH (10 mg/ml) to infarcted hearts. We compared this group with sham operated rats, CM in phosphate buffered saline (PBS), only PBS, and only GH-transplanted groups. Three weeks after transplantation, cardiac function was evaluated by echocardiography. The echocardiography confirmed that transplantation of 5×10^6^ CM with GH significantly improved cardiac systolic function, compared with the CM+PBS group (fractional area change: 75.1±3.4% vs. 60.7±5.9%, p<0.05), only PBS, and only GH groups (60.1±6.5%, 65.0±2.8%, p<0.05). Pathological analyses demonstrated that in the CM+GH group, CM were efficiently engrafted in infarcted myocardium (p<0.01) and angiogenesis was significantly enhanced (p<0.05) in both central and peripheral areas of the scar. Moreover, quantitative RT-PCR revealed that angiogenic cytokines, such as basic fibroblast growth factor, vascular endothelial growth factor, and hepatocyte growth factor, were significantly enriched in the CM+GH group (p<0.05). Here, we report that GH confined the CM effectively in infarcted myocardium after transplantation, and that CM transplanted with GH improved cardiac function with a direct contraction effect and enhanced angiogenesis.

## Introduction

Heart failure is the most notorious disease in developed countries. More than 20 million people suffer from heart failure all over the world [1]. Cardiac transplantation is the only radical treatment for severe heart failure, although the supply of donor hearts is not sufficient to cover the demand of transplant hearts [2]. In order to resolve this tight situation, basic science and clinical studies have focused on cell transplantation therapy [3]. A lot of clinical trials have been performed with several cell types, bone marrow stem cells, myoblasts, and cardiac progenitor cells in the last decade [4–6]. However, most of such clinical trials failed to show a markedly improved cardiac function [7]. Therefore, embryonic stem cells (ESCs) and induced pluripotent stem cells (iPSCs) were anticipated to overcome this unmet clinical need because of their high potential to differentiate into functional cardiomyocytes (CM) *in vitro* [8, 9]. Large-scale cell culture and purification systems for regenerative CM were recently established in the laboratory [10]. However, the low engraftment of transplanted CM has hampered the development of cardiac cell transplantation therapy [11, 12]. In order to recover the function of deteriorated hearts, transplanted CM must be engrafted efficiently. Hence, they need supportive materials. Biodegradable scaffolds have been investigated in cardiology. A variety of biocompatible material has recently been studied for regenerative medicine in the cardiovascular field [13, 14]. Gelatin hydrogel (GH) is a biodegradable water-soluble polymer gel that is generated by chemical cross-linking of gelatin [15]. Importantly, GH has already been available for clinical therapies with basic fibroblast growth factor (bFGF) to treat, for example, ischemic limb disease and Bell’s palsy [16, 17]. GH with bFGF also supported improvement of cardiac function after myocardial infarction (MI) [18]. However, the detailed mechanism of GH in improving cardiac function in cell therapies remains unknown.

Here, we report that GH enhanced the engraftment of transplanted CM and promoted angiogenesis by increasing the release of intrinsic angiogenic cytokines. Thereby, GH comprehensively ameliorated the function of infarcted hearts.

## Materials and Methods

### Assessment of coagulation of GH

In order to evaluate the thrombogenic properties of GH, blood was perfused over GH compound or type I collagen fibrils (Sigma-Aldrich, St.Louis, MO) at a wall shear rate of 1500 s^-1^ or 750 s^-1^. Platelet thrombi were visualized three-dimensionally in real time with confocal microscopy (Axiovert200; Carl Zeiss), as described previously [19].

### Isolation of fetal rat CM and MitoTracker staining

Ventricular CM were isolated from the hearts of 20-day-old fetal Lewis rats.

Before transplanting them, they were stained by MitoTracker (Invitrogen, Carlsbad, CA). MitoTracker was added to the medium for 10min and removed by centrifugation before collecting and transplanting CM.

### Cryoinjury and cell transplantation

Eight-week-old female athymic nude rats (Japan CREA, Japan) were used in this study. All protocols were approved by the KEIO University Animal Care and Use Committee. To standardize infarct size, MIs were induced by the cryoinjury method. The rats were anesthetized with a low dose of isoflurane. The anesthetized rats were ventilated with a rodent ventilator (Model SN-480-7, Shinano, Japan). A left thoracotomy was performed in the fourth intercostal space, and the pericardium was removed. Cryoinjury was induced with a metal probe (5 mm in diameter) cooled by liquid nitrogen. The cooled metal probe was applied to the left ventricle free wall for 30 seconds each time and applied five times. Fetal rat CM (5×10^6^ cells or 1x10^6^ cells) were transplanted with GH (10 mg/ml) one week after MI. We compared these groups with a sham group (only double thoracotomy), a group receiving CM with phosphate buffered saline (CM+PBS), a group receiving only GH, and a group receiving only PBS. Cell transplantation was carried out using a repeated thoracotomy and injecting the total bolus of cells into three separate injection sites, that is, the central cryoinjured lesion and the flanking lateral border zones.

### Echocardiography

Transthoracic echocardiography was performed 3 weeks after treatment (N = 10 each for PBS, GH, CM+PBS, CM+GH groups, and N = 5 each for sham, sCM+PBS, and sCM+GH groups where sCM indicates a small number of CM (1x10^6^ cells)). Rats were anesthetized with low-dose isoflurane for echocardiographic examination. Two-dimensional targeted M-mode traces were obtained at the papillary muscle level using an echocardiography system (Vevo 2100, Visual Sonics, Toronto, Canada). Left ventricular internal diameter in diastole (LVDd) and left ventricular internal diameter in systole (LVDs) were measured in at least three consecutive cardiac cycles. Ejection fraction, fractional shortening, and fractional area change were calculated with the Teichholtz formula.

### Histological and immunohistochemical analysis

After echocardiography, heart tissues were fixed in 10% buffered formaldehyde, embedded in paraffin, and sections were cut from the injured site at 1-mm intervals vertical to the long axis of the heart. Sections were selected at the middle of the injury site and stained with azan stain. Scar zones were evaluated as the ratio of azan-positive area to left ventricular area among PBS, GH, CM+PBS, and CM+GH groups. For immunofluorescence staining, heart tissues were fixed in 4% paraformaldehyde and embedded in an optimal cutting temperature compound, and then snap-frozen in liquid nitrogen. Micrometer-thick tissue sections were stained with antibodies against CM specific marker (troponin-T (Abcam, Cambridge, UK)) or endothelial cell (EC) marker (von Willebrand factor (vWF) (Abcam)) using anti-mouse immunoglobulin G antibody as a secondary antibody. Nuclei were stained with 4',6-diamidino-2-phenylindole dihydrochloride (DAPI) (Invitrogen). All of the *in vitro* images were acquired and analyzed with a fluorescence microscope (Axio Observer; Carl Zeiss Inc., Oberkochen, Germany). After immunofluorescence staining, transplanted CM were counted as troponin T and MitoTracker-double positive cells in the scar zone. Moreover, angiogenesis was evaluated by counting vWF-positive cells in the central and peripheral areas of the scar in PBS, GH, CM+PBS, and CM+GH groups. Densities of transplanted CM were compared between CM+GH and CM+PBS groups.

### Quantitative RT-PCR

After echocardiographic examination, heart tissue was obtained from the infarct area in individual rats and used for comparison between the two groups (N = 4 each). Total RNA was extracted using an RNeasy Mini Kit (Qiagen, Hilden, Germany), and cDNA was synthesized using the Superscript First-Strand Synthesis System (Invitrogen). cDNA was used as the template in a TaqMan real-time PCR assay using the LightCycler 96 System sequence detection system (Roche, Indianapolis, IN) according to the manufacturer's instructions. The specific primers and Taqman probes for vascular endothelial growth factor (VEGF) and bFGF, and hepatocyte growth factor (HGF) were Rn0511601_m1, Rn00570809_m1, and Rn00566673_m1. The data were normalized to glyceraldehyde-3-phosphate dehydrogenase (GAPDH).

### Statistics

All values are presented as the mean±SD. Statistical significance was evaluated using Student’s t test for comparisons between 2 mean values. Multiple comparisons between more than 3 groups were performed using an ANOVA, and significant differences between groups were evaluated using Tukey-Kramer test. A value of P<0.05 was considered significant.

## Results

### GH did not affect the thrombogenicity of the transplantation site

**Fig 1:**
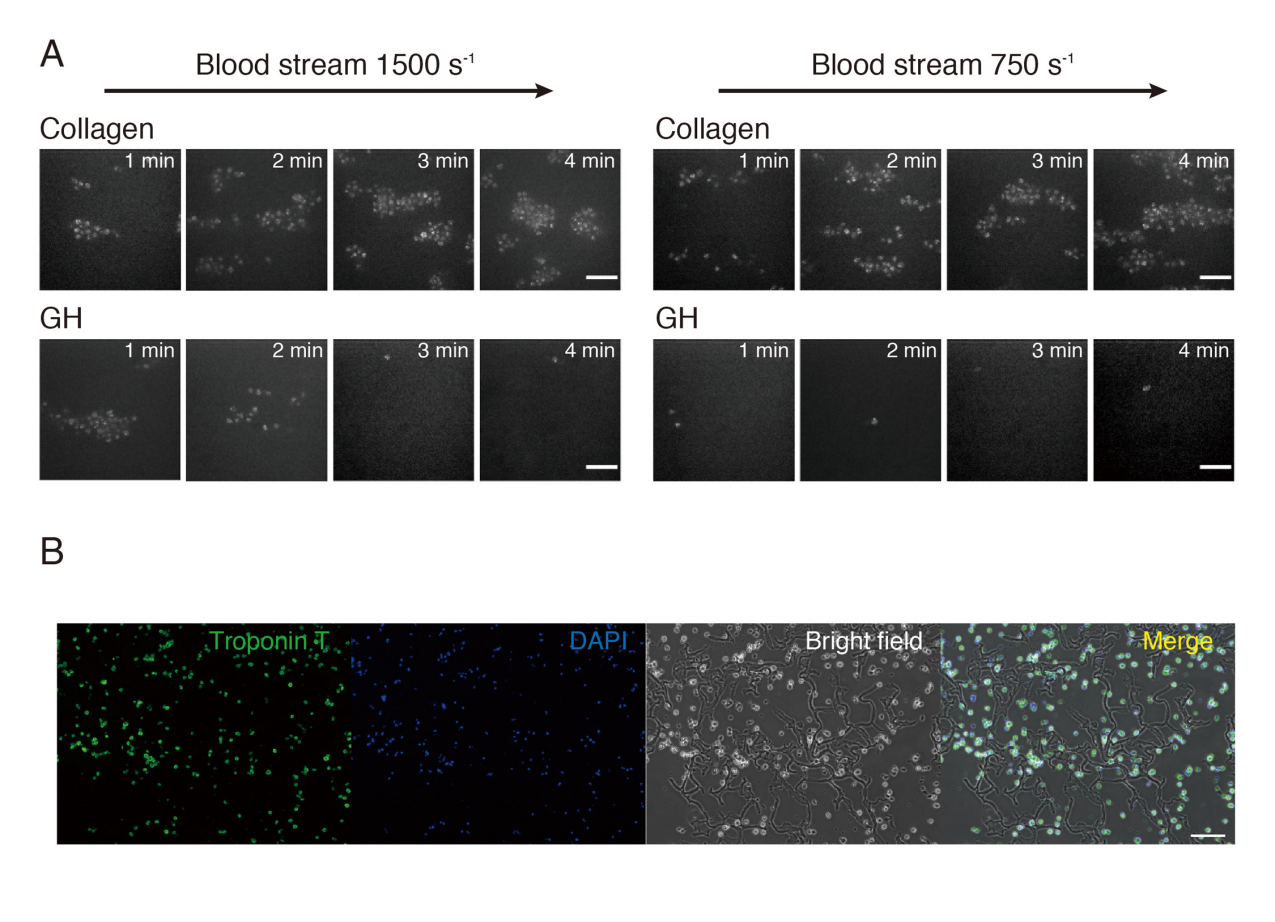
Thrombogenicity and cell adhesion of gelatin hydrogel (GH). (A) Thrombogenicity of gelatin hydrogel (GH) was compared with that of collagen. In a 1500-s^-1^ blood stream, non-specific aggregation took place in 1 to 2 minutes, but no aggregation was induced after 3 minutes. In a 750-s^-1^ blood stream, GH induced no aggregation at all. Bars are 10 μm. (B) Cardiomyocytes (CM) were premixed with GH before transplantation. CM were stained with cardiac troponin-T and 4',6-diamidino-2-phenylindole dihydrochloride (DAPI). Most of the CM were entwined with GH, and they were distributed evenly. Bar is 100 μm.

At first, we investigated the thrombogenicity of GH in comparison with collagen. If GH accelerated thrombogenicity too much, it would not be recommendable for the treatment of MI. In both 1500 s^-1^ and 750 s^-1^ blood stream conditions, collagen immediately induced the adhesion of platelets. However, GH showed only non-specific adhesion of platelets, and excess adhesion or three-dimensional thrombic growth did not occur even 4 minutes later (**Fig 1A**). The rat fetal CM were mixed with GH for cell transplantation. The mixture of CM and GH was stained with cardiac troponin T and DAPI to detect CM. Troponin T-stained CM were attached to GH (**Fig 1B**).

### Cardiac function improved after transplantation of CM with GH

**Fig 2:**
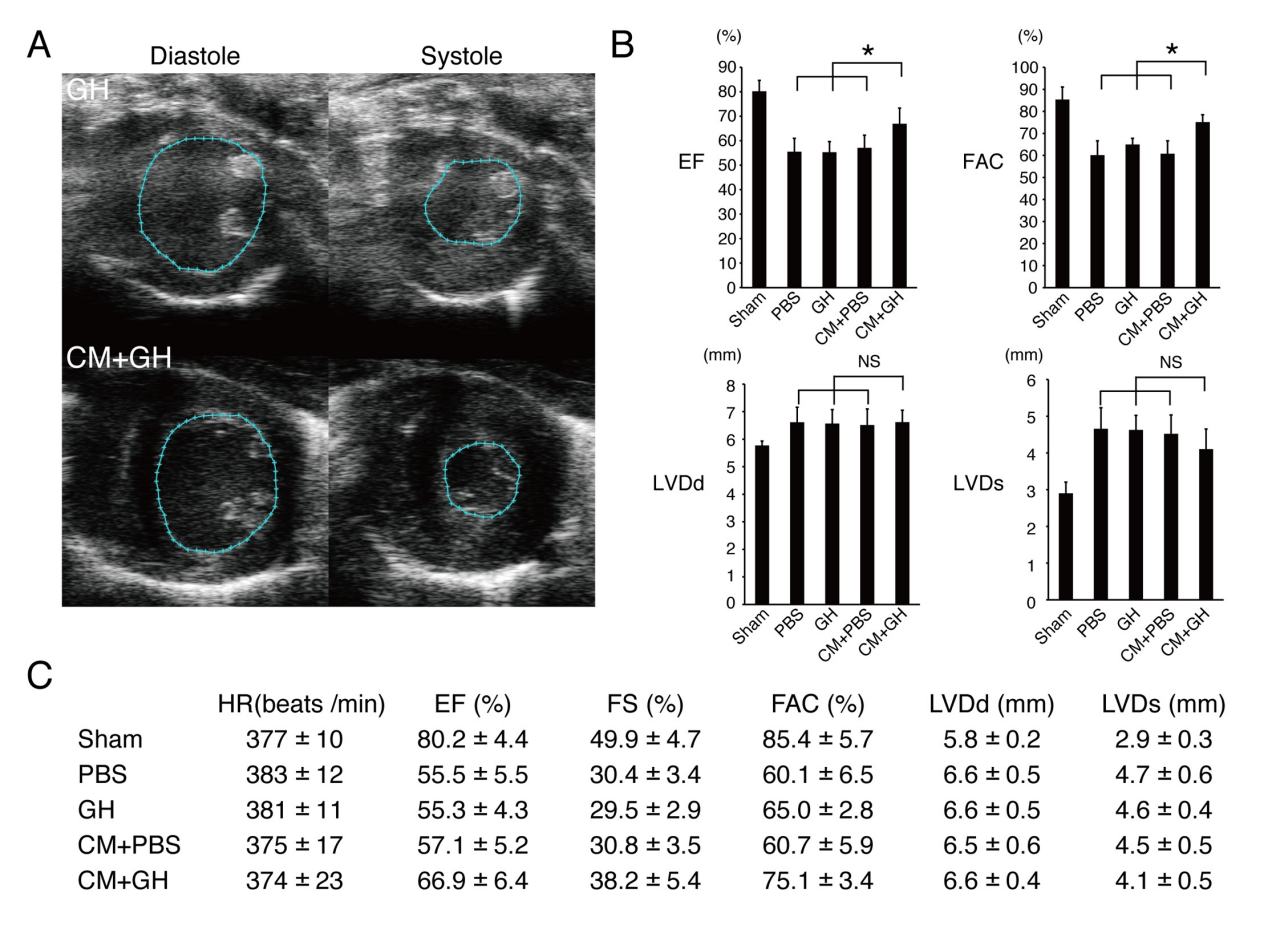
Transplantation of CM with GH improved cardiac function. (A) The representative figures of fractional area change (FAC) in the GH and CM+GH groups are shown. Only the CM+GH group showed better anterior wall motion. (B-C) Left ventricular systolic function was assessed by ejection fraction (EF), fractional shortening (FS), and FAC. All of them were significantly improved in the CM+GH group. (* P<0.05) Left ventricular internal diameter in diastole (LVDd) was elongated in all groups except sham group. Left ventricular internal diameter in systole (LVDs) was shorter in the CM+GH group. HR; heart rate.

Cardiac function after cell transplantation was analyzed by echocardiography (**Fig 2A–2C**). In fractional area change (FAC), GH and PBS groups showed a reduced anterior wall motion. The CM + PBS group also showed the infarcted anterior wall. Of note, the CM+GH group revealed a significantly restored cardiac function (**Fig 2A and 2B**). Cardiac systolic function was also evaluated by measuring ejection fraction (EF), and fractional shortening (FS). There was no significant difference in all parameters among the PBS, GH, and CM+PBS groups. Only the CM+GH group showed a significant improvement of cardiac systolic function (**Fig 2B and 2C)**. In order to evaluate the effect of CM number, a small number of CM (1x10^6^ cells; sCM groups) were also transplanted with either GH or PBS. They failed to recover cardiac function (**S1 Fig)**. LVDd of cryoinjured rats was dilated compared with the sham operation group. LVDs tended to be shorter in the CM+GH group than other groups (**Fig 2B and 2C**).

### Histological analysis of infarcted hearts

**Fig 3:**
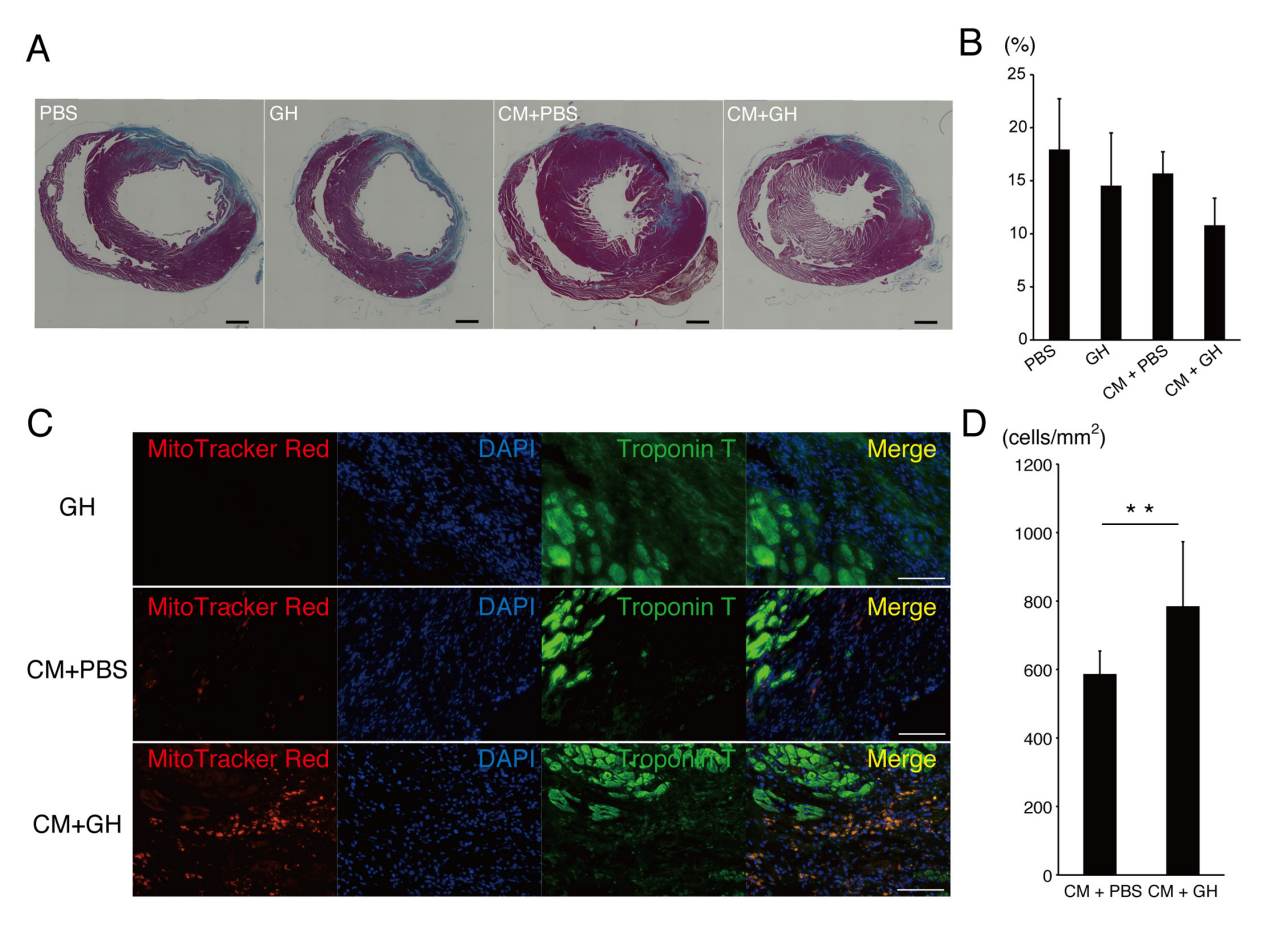
GH enhanced engraftment of CM. (A-B) Cardiac sections were stained with azan to evaluate the infarcted area. The CM+GH group tended to have a smaller infarcted area. Bars are 1 mm. (C) CM were prestained with MitoTracker-Red and sections were co-stained with cardiac troponin T and DAPI. In the GH group, there were no red signals in infarcted hearts. In the CM+PBS group, few CM were engrafted in the infarcted area. In the CM+GH group, more CM remained in the infarcted area. Bars are 100 μm. (D) The number of engrafted CM was increased significantly when transplanted with GH compared with CM transplanted with PBS. (** P<0.01)

All rats were sacrificed one month after MI, and their hearts were investigated pathologically. Hearts were dissected in papillary muscle level, and stained with azan staining. All hearts were dilated, except sham models, but the infarcted area in the CM+GH group tended to be smaller than in the other groups (**Fig 3A and 3B**).

### Engraftment of transplanted CM

In order to assess the mechanism for improving cardiac function after transplantation of the CM+GH group, the engraftment of transplanted CM was analyzed. CM were stained with MitoTracker-Red before transplantation and sections were observed with immunofluorescent microscopy. Autofluorescence of GH was not detected under immunofluorescent microscopy, but MitoTracker signals were clearly detected in infarcted myocardium (**Fig 3C**). The number of engrafted CM was compared between the CM+PBS and CM+GH groups. CM were significantly increased in the infarcted area of hearts transplanted with CM with GH (P < 0.01) (**Fig 3D**).

### GH enhances angiogenesis by increasing angiogenic cytokines

**Fig 4:**
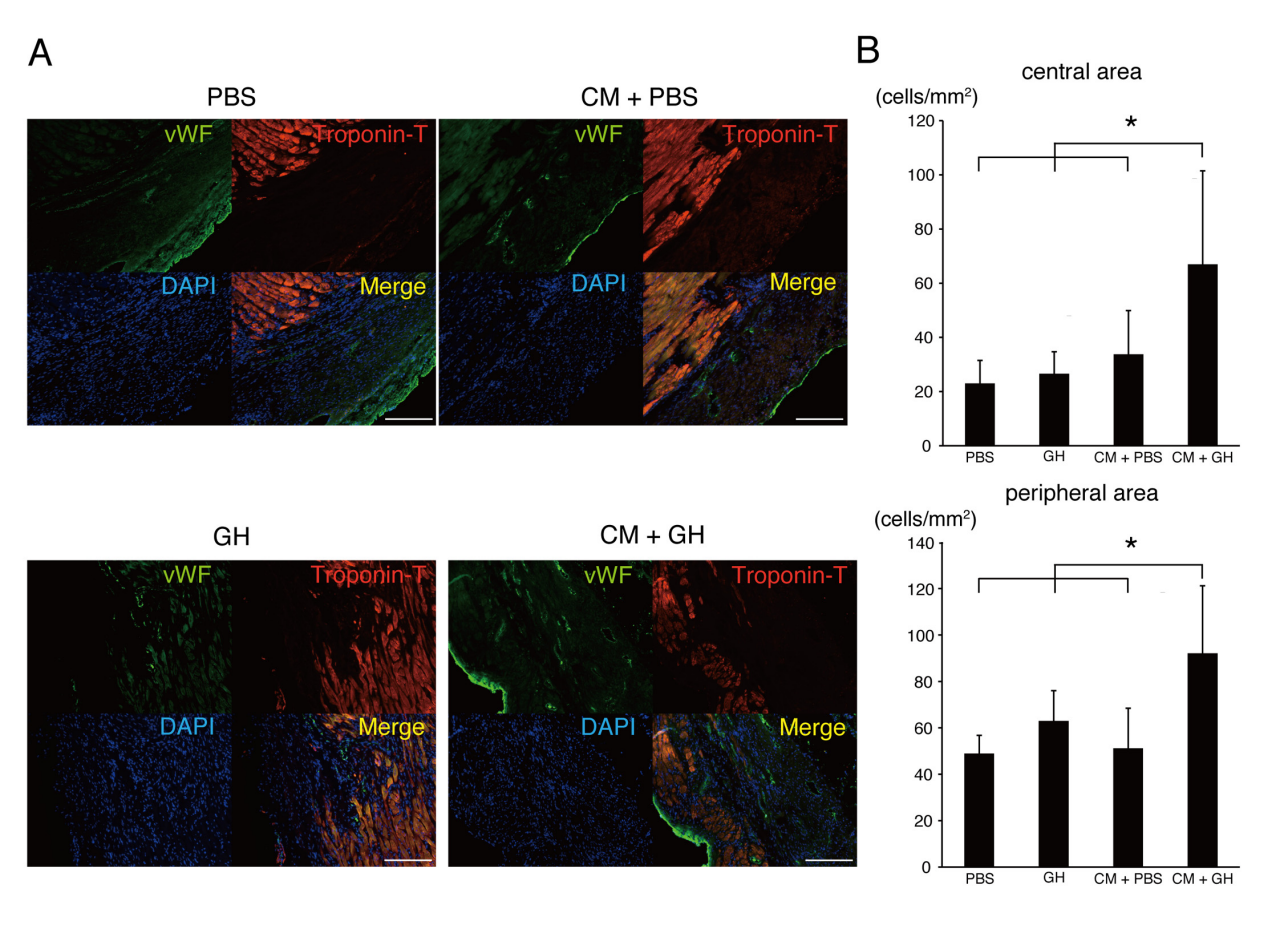
Transplantation of CM with GH increased newly formed vasculature. (A) Cardiac sections were stained with von Willebrand factor (vWF), and angiogenesis was evaluated in PBS, GH, CM+PBS, and CM+GH groups. The number of vWF-positive cells was increased in the CM+GH group. Bars are 200 μm. (B) vWF-positive cells were counted in the central and peripheral portions of the infarcted area. The transplantation of CM and GH significantly increased angiogenesis in both areas. (* P<0.05)

**Fig 5:**
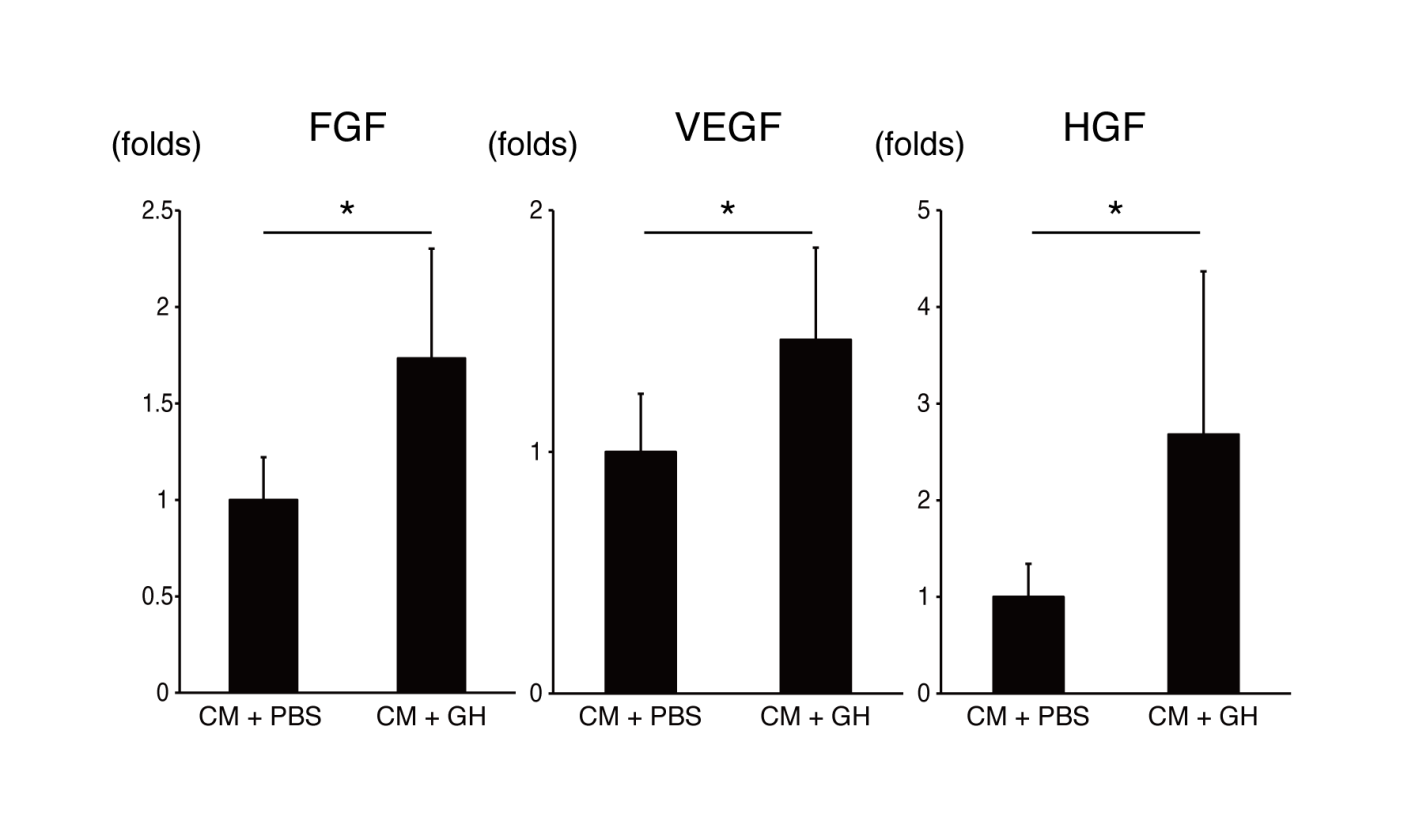
Transplantation of CM with GH increased the release of angiogenic cytokines. RNA was extracted from infarcted hearts, and angiogenic factors were evaluated. Basic fibroblast growth factor (FGF), vascular endothelial growth factor (VEGF), and hepatocyte growth factor (HGF) increased in the CM+GH group. (* P<0.05)

Neovascularization after cell transplantation was evaluated because it might affect cardiac function after MI. EC were stained with vWF in the PBS, GH, CM+PBS and CM+GH groups (**Fig 4A and 4B**). The number of vWF-positive cells (vascular cells) in the CM+GH group was significantly higher in both the center of the infarcted area and the infarcted border zone (**Fig 4B)**. Angiogenic factors promoting neovascularization were identified with quantitative RT-PCR. bFGF, VEGF and HGF were significantly increased in the CM+GH group, compared with the CM+PBS-transplanted group (**Fig 5**).

## Discussion

The co-transplantation of CM and GH successfully improved cardiac function by enhancing engraftment of CM and promoting angiogenesis. Cardiac regenerative therapies have been hampered by low cell engraftment and poor improvement of cardiac function in clinical studies [7]. As the most critical problem, it was assumed that somatic stem cells have very low potential to develop into CM both *in vitro* and *in vivo*. The regenerative CM from pluripotent stem cells, such as ESCs and iPSCs, were the hope of cardiac cell therapies. In order to obtain functional improvement, regenerative CM must be engrafted in infarcted hearts efficiently. However, transplantation strategies of regenerative CM have not been established yet. Because hearts beat and contract spontaneously unlike other organs, donor cells are easily drained from the transplantation sites. GH was reported to enhance the aggregation of cells and keep cells more vital [20]. This biodegradable material must have a positive effect on the engraftment of CM. As expected, the aggregated CM with GH persisted longer, and the sticky GH played a role in confining transplanted CM to the infarcted hearts. Moreover, GH did not enhance thrombogenicity even though gelatin is composed of collagen. This finding confirms that GH has no biological activity. This is a strong advantage for clinical application, because if it enhanced thrombogenicity, it would easily induce microvascular embolization, which would deteriorate infarcted hearts.

The number of CM in a heart was estimated to be 3x10^9^-1x10^10^ [21]. Thus, in contrast to other tissues, such as retina, a large number of CM will be necessary for cardiac cell therapies. Different doses of CM were assessed in this study. Clearly, the therapeutic effects of CM and GH depended on the cell number. Transplanting a smaller number of CM with GH could not recover the deteriorated cardiac function after MI. These data suggest that the number of transplanted CM is critical for combination therapy with CM and GH to improve cardiac function.

The functional improvement of infarcted hearts was also supported by significantly enhanced angiogenesis. In the qRT-PCR data, the representative angiogenic cytokines, VEGF, bFGF, and HGF, were markedly increased in hearts transplanted with CM in the presence of GH. VEGF is the major factor for vasculogenesis, and is expressed by EC. CM are also the major source of VEGF in the heart [22]. bFGF is known to be released from CM, EC, and fibroblasts [23]. It is a potent angiogenic factor and improves cardiac function via angiogenesis [24]. HGF is also released from EC and macrophages in infarcted hearts [25]. These cytokines are known to have synergistic effects on angiogenesis [26]. GH releases cytokines gradually, dissolves and completely disappears in a few weeks after transplantation *in vivo* [27], and it has already been used as cytokine releaser for bFGF [18]. These data strongly support that GH effectively absorbs released cytokines from transplanted CM and host heart, and sustainedly releases cytokines for a longer period, thereby enhancing angiogenesis. However, transplantation of only GH and CM+PBS failed to repair infarcted hearts, even though the CM+PBS group showed low numbers of engrafted CM. Consequently, the pleiotropic effects must be enhanced by co-transplantation of CM, and the humoral interaction between transplanted CM and a host heart must be augmented by GH to improve cardiac function.

In conclusion, GH strongly supported the engraftment of donor CM in infarcted hearts, and sustainedly released cytokines to promote angiogenesis. Thereby, the combination therapy of CM with GH could expand the possibilities of regenerative cardiac cell therapies.

## Supporting Information

S1 Fig Transplantation of small numbers of CM could not recover cardiac function. One million CM were transplanted with PBS or GH, but they could not improve cardiac function in comparison with the sole PBS or GH groups. sCM; small number of CM (1x10^6^ cells).
(TIF)
