# Supplementary figures and images for: Gelatin Hydrogel Enhances the Engraftment of Transplanted Cardiomyocytes and Angiogenesis to Ameliorate Cardiac Function after Myocardial Infarction
Source: PLoS One. 2015 Jul 17;10(7):e0133308. doi: 10.1371/journal.pone.0133308 (PMC4505846; doi:10.1371/journal.pone.0133308)

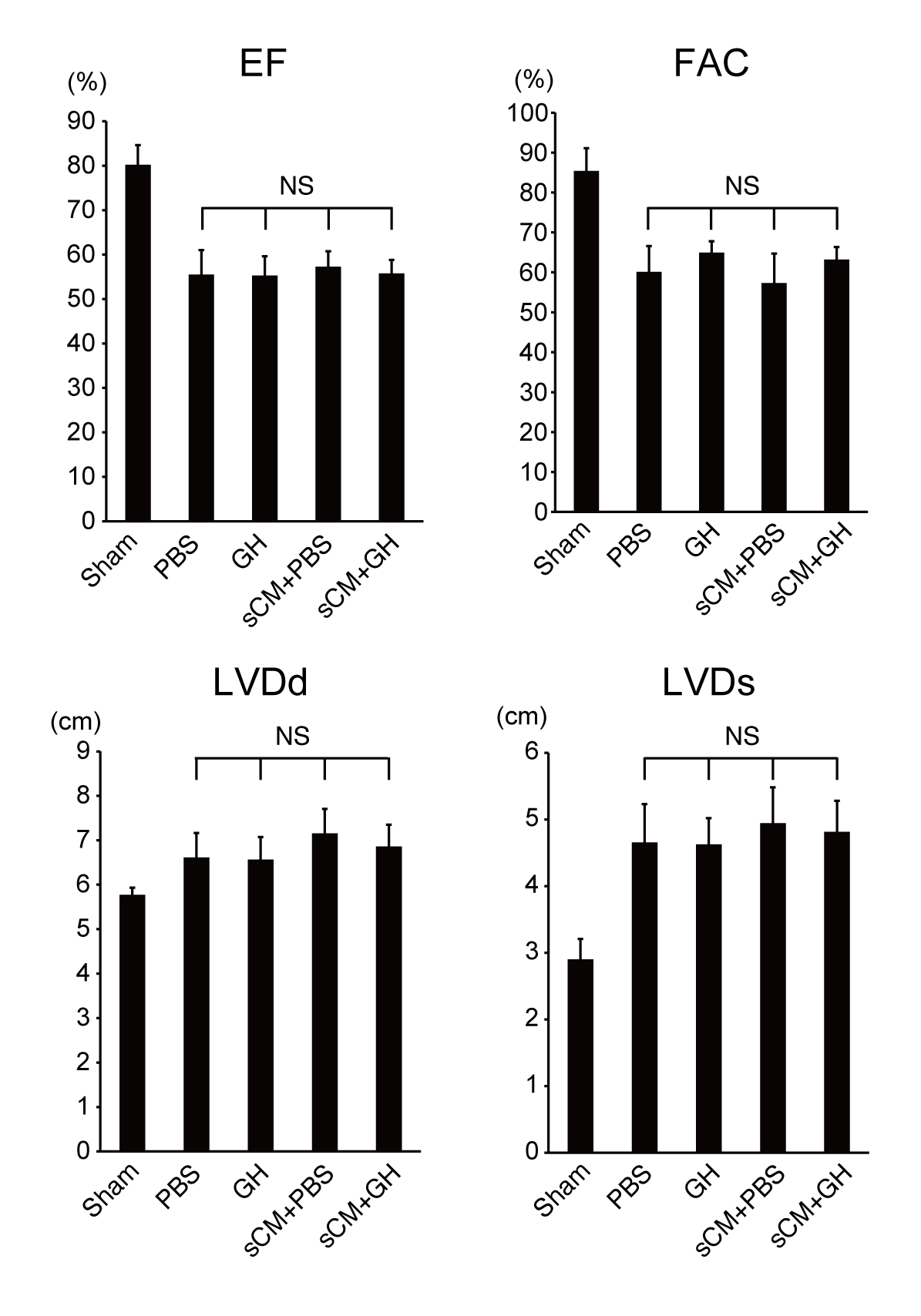

Supplement: S1 Fig — One million CM were transplanted with PBS or GH, but they could not improve cardiac function in comparison with the sole PBS or GH groups. sCM; small number of CM (1x10^6^ cells). (TIF) [file pone.0133308.s001.tif]
